# Shaping development by stochasticity and dynamics in gene regulation

**DOI:** 10.1098/rsob.170030

**Published:** 2017-05-03

**Authors:** Peng Dong, Zhe Liu

**Affiliations:** Howard Hughes Medical Institute, Janelia Research Campus, 19700 Helix Dr, Ashburn, VA 20147, USA

**Keywords:** gene regulation, development, gene regulatory network, bursting, gene expression noise, imaging

## Abstract

Animal development is orchestrated by spatio-temporal gene expression programmes that drive precise lineage commitment, proliferation and migration events at the single-cell level, collectively leading to large-scale morphological change and functional specification in the whole organism. Efforts over decades have uncovered two ‘seemingly contradictory’ mechanisms in gene regulation governing these intricate processes: (i) stochasticity at individual gene regulatory steps in single cells and (ii) highly coordinated gene expression dynamics in the embryo. Here we discuss how these two layers of regulation arise from the molecular and the systems level, and how they might interplay to determine cell fate and to control the complex body plan. We also review recent technological advancements that enable quantitative analysis of gene regulation dynamics at single-cell, single-molecule resolution. These approaches outline next-generation experiments to decipher general principles bridging gaps between molecular dynamics in single cells and robust gene regulations in the embryo.

## 1. Introduction

The development of a multicellular organism from a zygote takes place following a well-defined genetic blueprint. In the past decades, extensive studies based on a combination of biochemical, cell biological, genetic and genomic approaches have systemically characterized genetic players controlling development [1–3]. Collectively, these studies have depicted a static gene regulatory network (GRN) that governs diverse cellular behaviours such as cell proliferation, cell fate determination and morphological movements during animal development [4–7]. However, these conventional cell population-based endpoint assays were unable to reveal the spatio-temporal dynamics or the three-dimensional (3D) architecture of molecular systems operating in single live cells that eventually give rise to precise gene regulation during embryogenesis. Recent development of a set of advanced imaging tools for single-cell, single-molecule analysis have opened up exciting new opportunities to address these questions [8–11]. Here, we will first discuss the molecular origin of gene expression stochasticity and dynamics, and how these properties are harnessed at the systems level to control distinct cellular functions and developmental events. We will also review recent technological advances and pinpoint emerging directions for applying these new methods to decode gene regulation at different levels.

## 2. Stochasticity in gene regulation

Fundamental gene regulation steps such as transcription and translation are inherently stochastic processes. The stochasticity originates from intrinsic randomness of molecular dynamics and interactions in a living cell. The inherent stochasticity is largely averaged out by an increased number of mRNA or protein molecules within the reactant pool; nonetheless, under many circumstances it could propagate through the GRN and influence the functioning of genetic circuits, sometimes leading to the variability of cellular phenotypes during developmental processes.

### 2.1. Transcriptional and translational bursting

**Figure 1.**
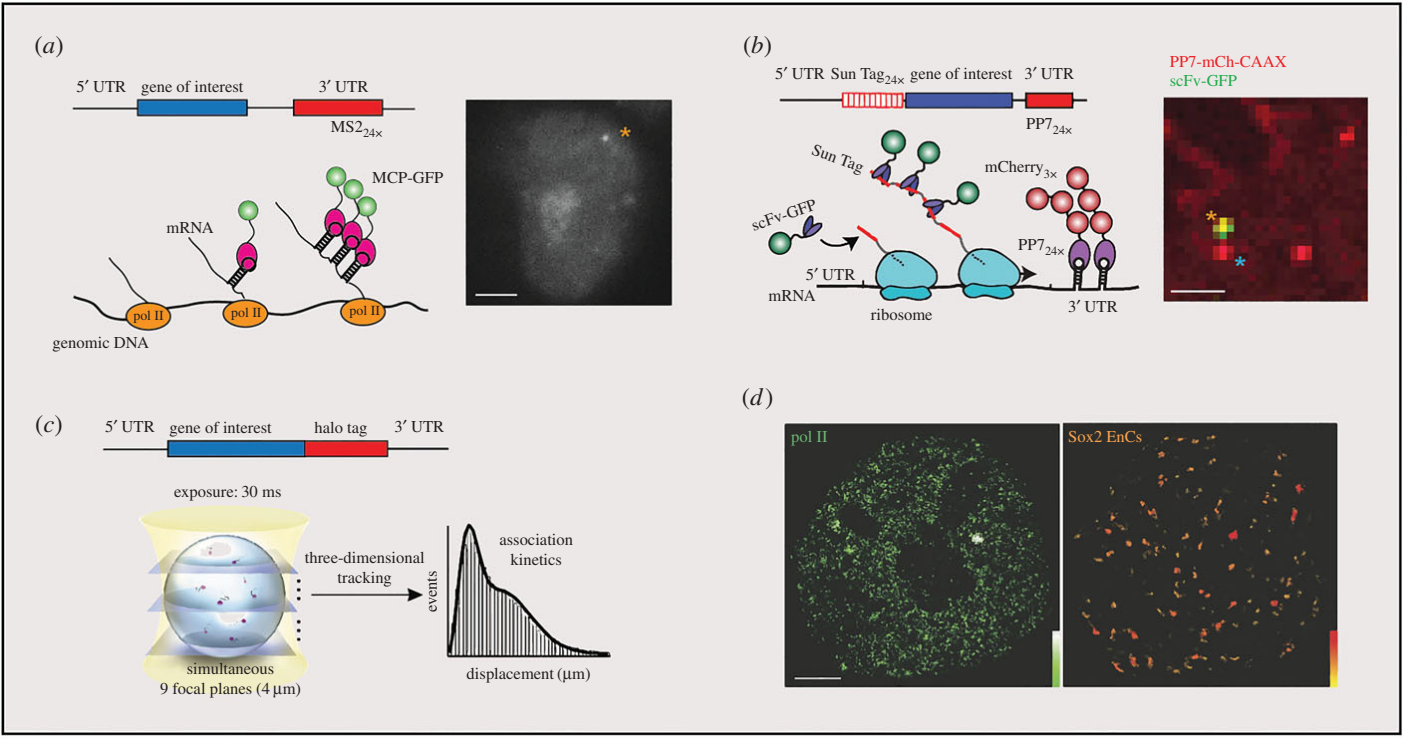
Single-molecule imaging reveals profound stochasticity in fundamental gene regulatory steps. (*a*) MS2 system used for imaging transcriptional bursting. A cassette of MS2 repeats was inserted into 3′ UTR of the gene of interest. When transcribed, the MS2 sequence forms stem loops that are bound by the fluorescent coat protein MCP-GFP. Yellow asterisk indicates the genomic locus of the labelled gene under transcription. (*b*) SunTag system used for imaging translational bursting. A reporter gene fused with a DNA fragment encoding 24 SunTag peptides is introduced into the cell, along with a second construct expressing a GFP-tagged single-chain intracellular antibody (scFv-GFP) that binds to the SunTag peptide with high affinity. In parallel, the transcribed mRNA molecules are labelled with the PP7 system (PP7-mCh-CAAX), which is fused to the mCherry fluorescent protein and also incorporates a plasma membrane-tethering domain (CAAX). Asterisks mark single mRNA molecules that are undergoing translation (yellow) or not (blue). (*c*) Fast three-dimensional tracking of TF movement by simultaneous multifocus microscopy (MFM). The displacement of moving single molecules is plotted as a histogram. (*d*) Live-cell two-dimensional single-molecule tracking reveals Pol II clusters (Dendra 2-Pol II, left) and Sox2 enhancer clusters (Sox2-Halo, right). The colour map marks local density. All scale bars, 2 µm. With permission, (*b*) is modified from reference [17], (*c*) is modified from [8] and (*d*) is modified from [18].

As the first step of gene regulation, transcription is shown to be a highly dynamic and stochastic event. The first evidence came from single-molecule fluorescent *in situ* hybridization (smFISH) experiments. smFISH allows quantification of mRNA molecules within a single cell, revealing extensive variation of mRNA copy numbers in individual cells both in culture and in tissues [12–14]. By using an MS2 or PP7 live-cell RNA labelling system, it is feasible to image transcription at single-molecule resolution and in real time [15,16]. These experiments uncover drastic characteristics of eukaryotic transcription whereby the production of new mRNA molecules from a gene occurs in a bursting manner (figure 1*a*) [19,20]. The bursting kinetics can be roughly described by two parameters—bursting size and frequency. The statistics of these two parameters are speculated to be regulated by different components of the transcription machinery such as transcription factor concentration, enhancer–promoter architecture, epigenetic environment, gene positioning and chromatin remodelling [21]. Most developmental regulators are thought to be transcribed in bursting kinetics [22]. Using the MS2 system, both Garcia *et al.* [23] and Lucas *et al*. [24] investigated the activation of the gap gene Hunchback by the gradient of the Bicoid protein in *Drosophila* embryo. They have both identified strongly induced bursting in the cells at the anterior pole but quite stochastic switching in those cells at the posterior pole [23,24]. In addition, *Nanog*, which safeguards embryonic stem (ES) cell ground state, shows drastic transcription bursting kinetics in mouse ES cells [25]. More interestingly, the frequency and duration of Nanog transcription bursts can be altered by switching cells from serum to 2i culture condition.

Protein translation from mRNAs also occurs in a bursting fashion. Several independent studies have recently demonstrated imaging of this fundamental biological process at the single-mRNA level in mammalian cells (figure 1*b*) [17,26–29]. Specifically, they labelled the mRNA transcript by using an MS2 or PP7 system and in parallel engineered the transcript to express a set of ‘SunTag’ [30] or ‘spaghetti-monster’ epitopes [31] that recruit multiple copies of fluorescent protein to monitor the production of the nascent protein. An interesting consensus from these studies is that different mRNA molecules within the same cell are translated at heterogeneous rates, suggesting that, at the single mRNA level, translation is a stochastic process.

### 2.2. Molecular dynamics underlying stochasticity

The stochasticity of gene expression originates from intrinsic randomness of molecular dynamics in living cells. Boiled down to the bottom, biochemical reactions involved in gene regulation, such as transcription, translation, epigenetic regulation and protein degradation, are all driven by a complex cascade of dynamic molecular interactions at the single-molecule level that usually involve multistep complex assembly and extensive interactions between protein and nucleic acids. Traditional experimental approaches for studying gene regulation mostly rely on measuring average mRNA or protein concentrations from a mixed cell population at a given time point, thereby lacking the ability to probe these dynamic events in living cells with high spatio-temporal resolution [32,33]. Rapid development of live-cell labelling chemistry and fast high-resolution imaging platforms provide unique opportunities to elucidate the physical reality of biochemical reactions in living cells [34–36]. Gebhardt *et al.* developed reflected light-sheet microscopy and firstly achieved single-molecule imaging of transcription factor (TF) binding to DNA in live mammalian cells [37]. This work was followed by Morisaki *et al*., who performed single-molecule imaging of HaloTag-tetramethylrhodamine (TMR)-labelled p53 and the glucocorticoid receptor [38]. Recently, Chen *et al.* developed a single-cell, single-molecule approach to image the binding of pluripotency factors (Oct4 and Sox2) onto target DNA and found that TF target search occurs following a trial-and-error sampling mechanism (figure 1*c*) [8]. Specifically, the binding between TF and enhancer was randomly interspersed by many rounds of non-specific TF–chromatin collision events. Statistically, the distribution of Sox2 residence times can be fitted by a two-component exponential decay model, in which the long-lived group corresponds to specific DNA-binding events, while the other group reflects non-specific binding, consistent with the results from imaging of early mouse embryos by photoactivatable fluorescence correlation spectroscopy (FCS) [39,40]. An important revelation from these results is that although individual TF–DNA-binding events are stochastic and nondeterministic, the overall statistics of TF–DNA binding dynamics (such as target site sampling frequency and average residence time) are highly sensitive to TF concentrations in the nucleus and the biophysical properties of the TF–DNA interaction.

Another emerging notion derived from recent single-cell studies is that the mammalian cell nucleus is highly compartmentalized, comprising heterogeneous function domains. For example, single-molecule imaging studies show that Sox2 target-binding sites form three-dimensional clusters that are spatially segregated from heterochromatic regions in single live ES cells [18]. More interestingly, the local diffusion and binding kinetics of Sox2 are differentially regulated within these clusters. Specifically, the Sox2 bound fraction is substantially increased inside clusters, consistent with higher local open chromatin concentrations and shorter three-dimensional diffusion times (τ_3D_) between stable binding events. The shortened τ_3D_ might provide a greater opportunity for recycling pre-assembled TF complexes and taking advantage of cooperative interactions between TFs on chromatin. Interestingly, these studies also suggest that even subtle changes in the position of target genes within individual clusters can lead to alterations in local target search features. For example, gene targets at the centre of the cluster can capitalize on different target search features relative to genes in the periphery of clusters. Thus, the local TF target search mode may be exquisitely modulated within distinct subnuclear environments and serve as an important mechanism for fine-tuning the rates of TF complex assembly at specific *cis*-regulatory elements. Complementary to these findings, Cisse *et al.* demonstrated that RNA polymerase II form diffract-limited, short-lived clusters in the live-cell nucleus [41]. It was further shown that the appearance of Pol II clusters predicts transcription bursting sites in living cells [42]. Extensive biochemical experiments established that binding of sequence-specific activators such as Sox2 to *cis*-regulatory elements precedes the Pol II transcription [6]. An emerging concept from many recent studies suggests that weak protein–protein interactions mediated by low complex (LC) domains are critical for dynamic molecular clustering [43]. Based on the fact that Sox2 stable binding-site clusters are extensively co-localized with Pol II clusters in the cell (figure 1*d*), it is tempting to speculate that these Sox2-enhancer clusters could serve as multivalent docking sites for dynamic recruitment of general transcription factors via weak protein–protein interactions. Such ‘clouds’ of weak multivalent protein–protein interactions would act as seeds triggering dynamic Pol II clustering and eventually lead to transcriptional bursting.

Collectively, these results suggest that molecular dynamics and architecture inside living cells serve as foundational mechanisms to generate and shape gene expression dynamics and stochasticity. With the rapid development of next-generation multicolour imaging modalities and new labelling strategies, it is important to further investigate how the three-dimensional genome organization specifically influences gene activity, and what gene products and mechanisms underlie the formation of these functional compartments in the nucleus.

## 3. Coupling stochasticity and dynamics in gene expression

The stochastic effects from fundamental steps of gene regulation will impinge and add onto the dynamic pattern of gene expression, resulting in temporal variation with stochastic fluctuations. The stochastic fluctuation, termed ‘gene noise’, occurs universally in microbial, single-cell eukaryotes and multicellular organisms that have developmental processes.

### 3.1. Gene expression noise originates from bursting

How does bursting contribute to gene expression noise? Pedraza & Paulsson presented a theoretical framework of the general quantitative relationship between bursting and gene expression noise [44]. Their analysis suggests that the random signals generated in one gene regulatory step have a deterministic effect on the following steps. Mathematically, the stochasticity originated from each regulatory step propagates within the GRN and is dampened by a coarse-grained time-averaging factor. This analysis together with a number of following studies pinpoint three important properties of dynamic and stochastic gene expression [14,21,33]: (i) gene expression noise originates from intrinsic stochasticity of regulatory steps such as transcriptional and translational bursts; (ii) noise can propagate through the GRN and spread to connected regulatory steps; and (iii) noise is averaged out after each signal amplification step by increasingly higher copy numbers of reactants, such as mRNA and protein molecules.

### 3.2. Gene regulatory network structure modulates gene expression noise

**Figure 2.**
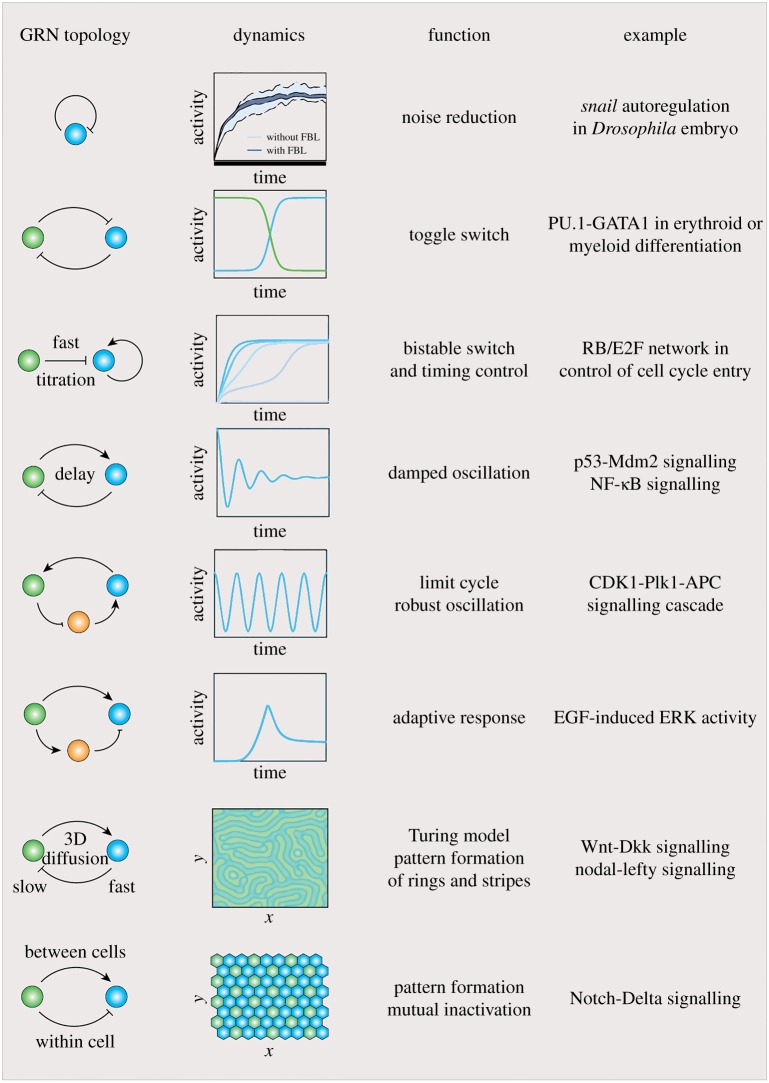
List of known GRNs with topology, dynamics, functions and examples. GRNs with defined topological structures are able to generate specific patterns of gene expression dynamics, including bistability, oscillation, adaptive response, noise regulation and diffusion in three-dimensional space, corresponding to different mechanisms for controlling cellular functions, cell fate decision and developmental patterning. References for examples: noise reduction [45]; toggle switch [46]; bistable switch and timing control [32]; damped oscillation [47,48]; robust oscillation [49]; adaptive response [50]; Turing model [51,52]; mutual inactivation [53].

Gene expression noise is regulated by GRN structure (figure 2). For example, the negative feedback loop can potently dampen gene expression noise, which might be necessary for precise control of gene expression levels [54–56]. This was first demonstrated by a synthetic auto-inhibitory reporter [45], but was later shown to exist in a variety of naturally evolved systems. One example is a developmental patterning gene *snail* in *Drosophila* embryo, which negatively autoregulates its own promoter and thereby maintains stable gene expression upon induction [56]. On the contrary, positive autoregulation amplifies noise [57,58]. Besides feedback loops by transcription control, microRNA-mediated incoherent feedforward loop has the capability of effectively reducing noise in parallel with fine-tuning protein levels [59]. Interestingly, theoretical analysis predicted that the noise buffering function depends on the inhibitory strength of microRNA and a moderate strength has optimal noise-reducing ability, yet these predictions remain to be validated experimentally.

Gene expression noise could also be beneficial under certain circumstances and thus does not always have to be suppressed. A recent study on NF-κB dynamics uncovered that the intrinsic noise within the GRN enhances the robustness of NF-κB oscillation in response to periodic tumour necrosis factor (TNF) signals. Specifically, the frequency variations of the NF-κB oscillation poise the cell population to respond to a broad range of dynamic stimuli, leading to efficient gene expression in dynamically changing environments [60].

### 3.3. Other factors that regulate gene expression noise

In parallel to GRN-mediated regulations, cells have evolved other mechanisms to control gene expression noise. For example, epigenetic modifications regulate noise through direct modulation of transcriptional bursting. Specifically, DNA methylation and histone deacetylation have been demonstrated to act as noise repressor, whereas histone acetylation does the opposite [61,62]. Interestingly, cellular compartmentalization was recently shown to function as a transcriptional noise filter through spatial partitioning of molecules in and out of the nucleus [63,64]. The nuclear retention of most transcripts is about twenty minutes, which is a similar time to scale transcription bursting or mRNA degradation and thus can efficiently average out the stochasticity of mRNA concentration by a factor of three to four [64]. The cost of this passive filtering is the loss of specificity in the spatial and temporal domains, yet it provides a general means of noise reduction for all the transcripts produced. Moreover, this interesting mechanism might have general implications in the evolutionary advantage of sophisticated cellular organizations in higher eukaryotes.

## 4. Gene expression dynamics at the systems level

Countless gene regulatory events take place within a single mammalian cell, driving dynamic expression of thousands of genes. The products of these genes functionally interact with each other in real time via feedback loops (figure 2), forming a number of interconnected circuits within the whole GRN network [3,65].

### 4.1. Gene regulatory network structure determines gene expression dynamics

From a physicist's point of view, if we know the kinetic parameters describing each regulatory event and the topological structure of the GRN, we should be able to formulate a set of differential equations to quantitatively describe the expression dynamics of any gene within the network. The variable for each equation corresponds to the expression of a particular gene as the function of time. The solution for each equation represents the thermodynamic evolution for a single gene product, and the solutions for the whole set of equations should describe the dynamic motion of the entire system. Depending on the parameter space, dynamic systems formulated by differential equations are able to generate diverse and interesting behaviours such as adaptive response, pulse, bistability, oscillation and chaos (figure 2) [49,50,66]. Meanwhile, the ‘one-to-one’ relationship between equations and solutions suggests that understanding GRN functionality requires precise measurement of gene expression over time.

### 4.2. Gene regulatory network shapes temporal dynamics

The relationship between gene expression dynamics and the GRN topological structure has been studied for over a decade by both ‘top-down’ network analysis and ‘bottom-up’ network engineering [3,65,67]. One emerging property in the GRN is the prevalence of bistability in the system (figure 2). Generally speaking, bistability means that the output of a system has two stable equilibrium states. Bistability is critical for biological systems, because it is required for generating digital and switch-like behaviours such as cell fate determination. Gardner *et al.* first built up a simple gene circuit comprising mutual transcriptional repression and showed that this genetic toggle switch can robustly generate bistability in gene expression [46]. Bistability also exists in natural systems. For example, the autoregulation of E2F transcriptional factor coupled with its ultrafast sequestration by the Rb protein generates bistability, dictating the switch of cell cycle between quiescence and proliferation [32].

Another dynamic feature in the GRN is oscillation, which is important for regulating periodic cellular processes such as cell cycle and circadian rhythm [49,68–70]. Although a two-node module, such as mutual repression with time delay, is able to generate damped oscillation, a three-node module is the minimum set-up for robust oscillation cycles (figure 2) [49]. Rust *et al.* probed the origin of circadian oscillation in cyanobacteria, and found that a small network comprising coupled positive and negative feedback loops maintains synchronized oscillation per day for several weeks [70]. Interestingly, several oscillation circuits can be coupled with each other to execute more complex regulations [71]. The GRNs governing mammalian circadian oscillation and the cell cycle could be even more intricate in terms of the number of genes and the degree of feedback/feedforward loops involved.

In higher eukaryotes, the integration of epigenetic regulations into the GRN provides more controls to gene expression dynamics. For example, Bintu *et al.* found that distinct types of epigenetic modifications, such as DNA methylation, histone deacetylation and histone methylation, have different effects in shaping gene expression dynamics [72]. Specifically, although all these modifications lead to transcriptional repression, they work at different time scales and thereby generate distinct temporal kinetics of epigenetic memory.

### 4.3. Gene regulatory network orchestrates spatio-temporal dynamics

The cell positional information has to be taken into account as a parameter for modelling gene expression during animal development, particularly for the case dealing with spatio-temporal distribution of morphogens. In this scenario, the dynamic evolution of a system can be formulated by a set of partial differential equations, which describe the variation of variables as a function of both time and space. The GRNs represented by partial differential equations are capable of generating molecular gradients within three-dimensional space (figure 2). For example, Cao *et al.* explored a synthetic circuit that forms self-organized core-ring patterns and showed that the ring width scales with the colony size, suggesting a self-controlled scaling mechanism dictated by the GRN structure [73]. In cultured mammalian cells, Sorre *et al.* monitored the expression dynamics of Smad4 protein as well as the transcriptional activity of Smad3 at the single-cell level to investigate the response of the GRN to different types of ligand stimuli [74]. Their results suggested that a TGF-β-mediated GRN responds to ligand stimuli in an adaptive mode. Specifically, high-frequency pulsed stimulations result in higher output than that from a mono-phase, sustained input, which serves as a mechanism for accelerating cell fate decision by morphogen gradients.

## 5. Shaping developmental processes by stochastic and dynamic gene expression

Animal development is a highly regulated spatio-temporal process in which cells undergo lineage commitment, proliferation and migration, giving rise to patterned tissues and organs. The amplification of cell number is accompanied by increasingly more complex organization and patterning of different cell lineages. Although the whole developmental process is regulated by numerous genes, a specific event may only heavily rely on a couple of regulators, consistent with a hierarchical topology of the developmental GRN [1,75].

### 5.1. Cell fate plasticity

The intrinsic stochasticity of gene regulatory events results in heterogeneous gene expression in single cells. This is not always deleterious and could be very useful for generating a repertoire of cells with plastic identities. The cell fate plasticity has been extensively studied by emerging single-cell transcriptome profiling technology (reviewed in [76–78]). By using a single-cell droplet-barcoding RNA sequencing approach, Klein *et al.* revealed that when the leukaemia inhibitory factor (LIF) was withdrawn from the culture medium, ES cells follow highly heterogeneous differentiation processes, indicated by clustered gene expression profiles in high-dimensional space [79]. Interestingly, in a small fraction of cells, pluripotent factors maintain high expression levels and epiblast markers do not appear until 7 days after LIF withdrawal. Single-cell heterogeneity was also investigated during haematopoiesis. By labelling and sequencing of individual haematopoietic progenitor cells, Perie *et al.* and Paul *et al.* showed that haematopoietic stem cells are not an equipotent, self-renewing pool but are a mixed population with differentiation bias towards various lineages [80,81]. The heterogeneity in gene expression among cells provides a ‘bet-hedging’ strategy that can prime pluripotent or multipotent stem cells to rapidly respond to a range of developmental cues. In addition to its role in cell fate plasticity, this strategy has also be shown to be important for facilitating bacteria to adapt to different growth environments for the purpose of survival [82].

### 5.2. Cell fate decision

Although noise generates cell fate plasticity within a cell population, a committed cell has one unique trajectory for gene expression and differentiation. Therefore, measuring expression dynamics of master cell fate regulators (such as TFs) would provide valuable information regarding how the GRN regulates cell fate decision. For example, combining network analysis and the measurement of E2F transcription dynamics at the single-cell level, Dong *et al.* uncovered that the network structure modulates E2F dynamics to generate bistability for coordinating two distinct functions—the control of the probability of cell-cycle entry by Myc and the control of cell-cycle pace by G1 cyclin/cyclin-dependent kinases (CDKs) (figure 2) [32]. This research highlights the fact that a naturally evolved system might have complex GRN structure to achieve multitask control of cellular processes. On the other hand, this study also addressed the question about how cell-cycle length is controlled, opening new opportunities for studying the relationship between cell cycle and lineage commitment.

One hallmark of cell differentiation is the increase of cell-cycle length, in particular the G1 phase [83,84]. For example, in the mouse nervous system, cell-cycle length in the ventricular zone increases from 8 h at the onset of neurogenesis to up to 18 h at the end [85]. Conversely, overexpression of G1 cyclin/CDKs or loss of CDK inhibitors, such as p27, shortens cell-cycle pace and impairs neurogenesis [86–88]. These findings suggest that the complex cell fate decision during differentiation is likely to be a synergistic effect from a GRN controlled by master cell-fate and cell-cycle regulators. As a result, measurement of the dynamics of lineage-specific TFs at different cell-cycle stages might provide key insight into understanding the cell fate decision process. Indeed, Kueh *et al*. measured the expression dynamics of PU.1 during myeloid differentiation and found that, in individual cells, the accumulation rate of this central regulator remains constant, while its final concentration varies according to the cell-cycle length [89]. Mathematical modelling revealed that the overall GRN comprising PU.1 and the cell-cycle network allows the system to switch between one undifferentiated state and two alternative differentiated states. This study exemplifies an avenue for studying similar types of questions.

### 5.3. Developmental patterning

GRNs that regulate morphogenesis have much more complex structures, which include not only regulators of cell division and fate but also components governing intercellular communication, such as Wnt, TGF-β superfamily, Notch, FGF and Hedgehog pathways [2,3,90]. The interconnected network topology are able to coordinate dynamic expression of a cohort of intracellular proteins, as well as some small secretory ligands that diffuse within three-dimensional space to control the patterning of different tissues. For example, during *Drosophila* embryo development, the Bicoid protein molecules are synthesized at the anterior pole and diffuse along the embryo axis, forming an exponentially decreased gradient [91]. This gradient then triggers compartmentalization for four target gap genes that establish the initial body segmentation. Although the Bicoid gradient was demonstrated to be essential, one important unsettled issue in this field is whether the Bicoid gradient alone is able to generate the pattern with such stunning precision, or whether it requires additional regulations, regarding the inevitable stochasticity in gene expression. Through mathematical modelling of the GRN, Manu *et al.* suggested that the cross-talk among gap genes is sufficient to reduce the patterning variance, though this prediction needs to be validated by more experiments [92,93].

Apart from morphogen gradient-dependent control, a few patterns such as rings or stripes can also be shaped within three-dimensional space by specific GRN structures. One well-studied example is the Turing model [94,95]. This model considers a reaction–diffusion mechanism in a simple GRN of two nodes—A and B. A diffuses slowly and produces B, whereas B diffuses rapidly but inhibits A (figure 2). Thereby, as the process initializes, the fast-diffusing B suppresses A at its surrounding regions, resulting in the spontaneous formation of rings and strips along the diffusion axis. The Turing model-defined structure has been identified in many developmental GRNs. For example, during murine hair follicle formation, the Wnt ligand was shown to serve as a short-range activator and the Dkk protein as a long-range inhibitor. The dynamic interplay between this pair of genes thus fits the Turing model and was indicated to determine the hair follicle spacing [51]. Another example is in mesendoderm formation, where a module comprising Nodal and its antagonist Lefty was speculated to function in a similar way to define the mesendoderm territory and to prevent its expansion into the ectoderm [2,52].

Differing from the reaction–diffusion Turing mechanism, the Notch-Delta GRN regulates multicellular patterning via direct cell to cell contacts [53,96]. Specifically, the Delta ligand trans-activates Notch in neighbouring cells, while *cis*-inhibiting Notch in its own cells through different configurations of molecular interactions. Mathematical modelling of the GRN suggested that the network is able to generate mutual-inactivation dynamics between Notch and Delta in the same cell, leading to an ultrasensitive switch between mutually exclusive signal sending (high Delta/low Notch) and receiving (low Delta/high Notch) states [53]. This mutual inactivation can amplify small differences in ligand concentration among neighbouring cells and facilitate cell fate decision and pattern formation.

## 6. Advances in imaging technology for probing dynamic and stochastic gene expression

### 6.1. Imaging molecular dynamics in gene regulation

**Figure 3.**
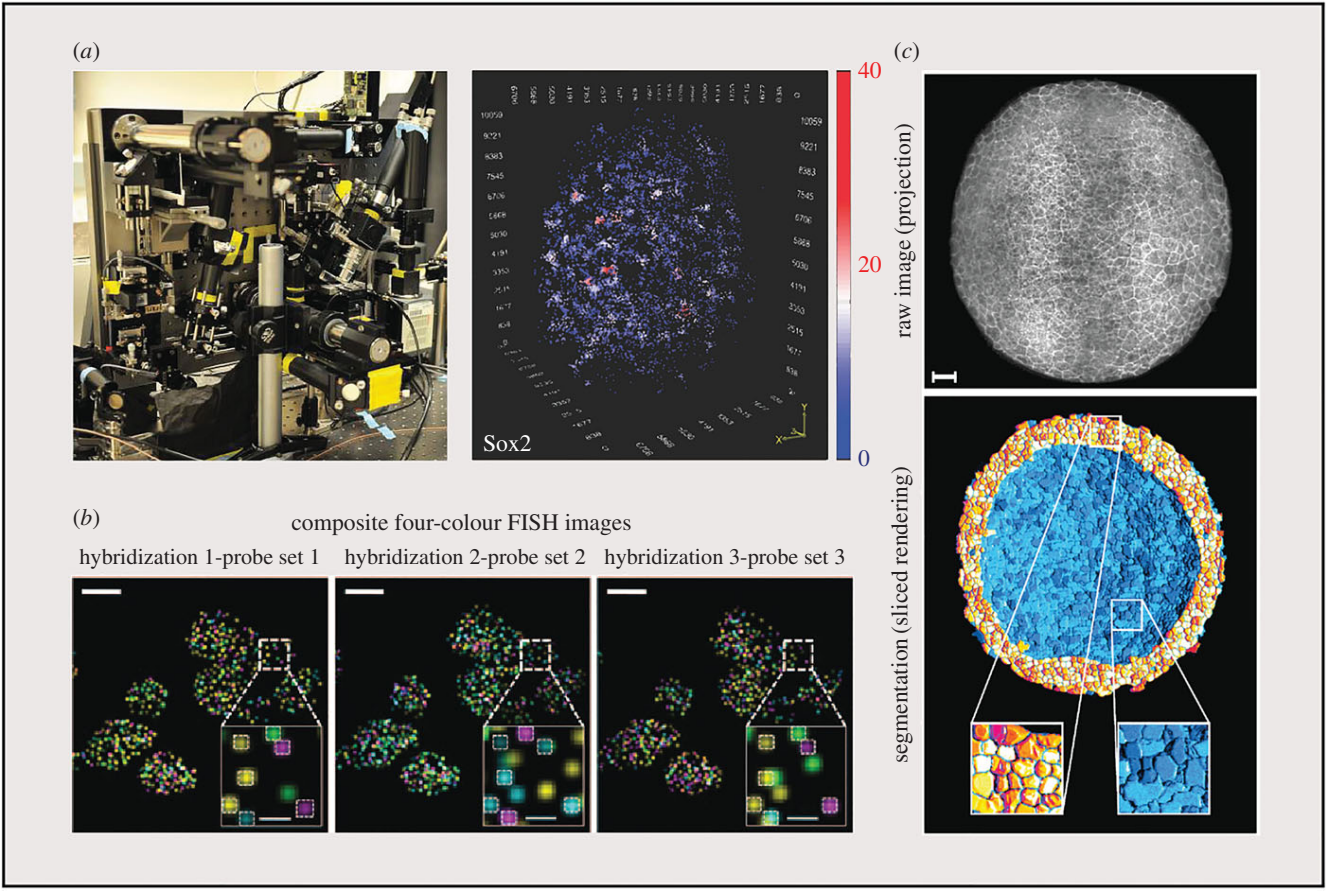
Imaging technology for probing dynamic and stochastic gene expression. (*a*) Imaging of the 3D Sox2 cluster by using a lattice light sheet scope. Left panel, photograph of an assembled lattice light sheet scope; right panel, three-dimensional density map of a Sox2 cluster in a single ES cell nucleus. (*b*) Composite four-colour FISH data from three rounds of hybridizations on multiple yeast cells. Genes are encoded by multiple rounds of hybridization using different probe sets. The boxed regions are magnified in the bottom right corner of each image. Spots co-localizing between hybridizations are detected and have their barcodes extracted. Spots without co-localization are attributed to non-specific binding. Scale bar, 5 µm. (*c*) Three-dimensional raw image projection (up) of zebrafish embryos (6 h post fertilization) expressing fluorescent markers labelling all membranes and segmentation results of sliced embryo (bottom). Scale bar, 50 µm. Panel (*a*) is modified from reference [18,35], (*b*) is adapted from [102] and (*c*) is modified from [103], with permission.

Modelling and analysing different GRNs underlying developmental control are limited by the precision of constructed transfer functions which describe the input–output relationship of fundamental gene regulatory steps, such as transcription and translation. However, great challenges exist for increasing precision, because every step dictates a complex cascade of dynamic molecular interactions. For example, a single transcription step includes the binding of TFs onto enhancers or promoters, the assembly of pre-initiation complex, elongation and termination in parallel with chromatin remodelling events such as nucleosome remodelling and epigenetic modifications [97]. Moreover, components in this machinery work with high-order cooperativities, bringing in more complexity for determining the physical nature of these biochemical reactions [98]. Conventional approaches such as biochemistry and structural biology provide little information about *in vivo* kinetics and stochasticity, and thus cannot resolve this layer of regulation, whereas imaging provides a unique opportunity by directly observing these dynamic processes in real time. Recent improvements in chemical dyes and fast high-resolution imaging platforms have allowed the direct labelling of single molecules and the tracking of their binding, dissociation and diffusion dynamics in live cells [11,34,99,100]. For example, we can directly image the binding of a TF or other DNA-binding protein at its genomic *cis*-regulatory elements and calculate its resident time [8,101]. Moreover, improved aberration-corrected multi-focus microscopy (MFM) can generate an instant stack of images from nine focal planes, opening an avenue for high-resolution three-dimensional imaging of single-molecule dynamics in real time [36]. Importantly, the newly developed state-of-the-art lattice light sheet scope enables the painting of a three-dimensional molecular interaction density map of TF within a single cell (figure 3*a*) [18,35]. These emerging techniques for non-invasive high-resolution imaging thereby provide us with effective tools for accurately measuring spatio-temporal dynamics of molecular systems in live cells at the single-molecule level.

### 6.2. Imaging gene expression heterogeneity within cell population

Several imaging techniques have been developed recently for measuring gene expression heterogeneity within a heterogeneous cell population or a tissue. One promising approach is smFISH, which can quantitatively determine the number of RNA molecules in individual cells. This approach has been continuously optimized with probe barcoding and sequential hybridization to enable multiplexed quantitative profiling of hundreds of genes at the single-cell level (figure 3*b*) [13,102]. It has been applied for analysing embryonic stem cell pool as well as three-dimensional mouse hippocampus, revealing novel cell identities within different regions of the tissue [62,104]. Recently, this technique was improved for cell lineage tracing. The principle is to create barcoded recording sequences that can be integrated into the genome and altered by CRISPR/Cas9-targeting mutagenesis during cell division. Finally, the lineage information will be read out from altered recording sequences through multiplexed RNA smFISH [105]. However, the smFISH experiment can only be performed on fixed samples, limiting its ability for resolving the information of gene expression at the temporal scale.

### 6.3. Imaging gene expression dynamics at the single-cell level

At the systems level, monitoring gene expression dynamics over time at single-cell resolution is critical to understanding cell fate decision and the functionality of the GRN. One common strategy to achieve this goal is via long-term imaging of cultured live cells with integrated fluorescence biosensors [106]. Recent technological advances from several aspects have greatly expanded the application of this approach, in particular for mammalian cells. The first advance comes from the design and generation of biosensors. Depending on the layer of dynamics chosen for monitoring, a cassette of fluorescence protein coding sequence can be placed downstream from promoters for detecting transcriptional dynamics, or directly fused to the protein of interest for capturing protein concentration dynamics [107]. In other cases, fluorescence resonance energy transfer (FRET) biosensors with phosphorylation-responsive elements have been used for probing the activity of protein kinases [108]. A remarkable improvement was made by Regot *et al*., who engineered the responsive domain of kinase substrate to convert phosphorylation into localization changes, providing a general approach for rapidly generating reporters for protein kinase activities [109]. Moreover, the development of revolutionary CRISPR/Cas9 genome editing tools have enabled efficient construction of reporter cell lines with knock-in alleles that can faithfully reflect gene regulation within the native chromatin [110,111]. The second advance comes from the improvement of imaging platforms. Although the wide-field epi-illumination scope is still sufficient for recording intensity-based dynamic signals, more advanced platforms such as the wide-pinhole confocal microscope, light-sheet microscope, two-photon microscope and adaptive optics are available for imaging more challenging samples and can generate images with increased resolution and volumes [106,112]. One of the breakthroughs in live imaging during the past decade is the development of state-of-the-art light-sheet microscopy platforms for imaging the developmental process of whole embryo at high spatio-temporal resolution for long periods of time (figure 3*c*) [10,100,113].

One emerging challenge accompanying the advancements in microscopy is that the ‘big data’ acquired by these high-resolution, fast-imaging platforms require convenient and efficient computational tools for data storage, management and imaging analysis. Many open-source or commercial software programs are currently available to address specific imaging analysis steps such as cell segmentation, tracking, signal quantification and clustering [103,114,115]; however, none of these programs can provide a complete set of solutions. The automation of certain steps, in particular cell tracking, remains an intimidating challenge because of the difficulties in tracking fast-moving cells or identifying cells with dramatic morphological change during cell division. A bright future in the field will be to integrate automated imaging platforms, programmable microfluidic-guided cell sorting and endpoint single-cell genomic sequencing techniques to probe gene regulation at the single-cell level with both temporal dynamics and whole-genome coverage.

## 7. Concluding remarks

Over the past decades, extensive genetic and biochemical studies have mapped out complex pathways and interactions that connect individual regulatory elements to a hierarchical GRN. However, a central remaining question is how the GRN operates in living systems, eventually giving rise to the precisely ordered execution of developmental programmes. To address this problem, we need to understand dynamic gene regulation at both molecular and systems levels. On the one hand, dissecting molecular dynamics at the single-molecule level will uncover the biophysical principles governing fundamental gene regulatory processes. Specifically, this may lead to the delineation of exact roles of each gene regulatory layers, including site-specific TFs, epigenetic regulators, housekeeping transcription machinery and the three-dimensional chromatin architecture. On the other hand, measuring gene expression dynamics at the single-cell level will reveal the information processing framework underlying the GRN. This may not only delineate the control logic underlying different core GRN structures governing cell fate determination and tissue morphogenesis, but also might uncover the overall organizing principles that coordinate the transition through different developmental stages. Finally, mathematical modelling will integrate the knowledge gathered from different perspectives to reconstruct a quantitative and comprehensive view of how gene regulation orchestrates the spatio-temporal choreography of development.
